# Astrocytoma-associated antigens - IL13Rα2, Fra-1, and EphA2 as potential markers to monitor the status of tumour-derived cell cultures *in vitro*

**DOI:** 10.1186/s12935-014-0082-z

**Published:** 2014-08-22

**Authors:** Monika Witusik-Perkowska, Magdalena Zakrzewska, Malgorzata Szybka, Wielislaw Papierz, Dariusz J Jaskolski, Pawel P Liberski, Beata Sikorska

**Affiliations:** 1 Department of Molecular Pathology and Neuropathology, Medical University of Lodz, Czechoslowacka 8/10 str, Lodz, 92-216, Poland; 2 Department of Pathomorphology, Medical University of Lodz, Czechoslowacka 8/10 str, Lodz, 92-216, Poland; 3 Department of Neurosurgery, Medical University of Lodz, Kopcinskiego 22 str, Lodz, 90-153, Poland

**Keywords:** IL13Rα2, Fra-1, EphA2, Astrocytomas, Cell culture

## Abstract

**Background:**

The molecular heterogeneity of high-grade astrocytomas underlies the difficulties in the development of representative and valuable *in vitro* experimental models for their studies.

The purpose of our study was to estimate the value of astrocytoma-associated antigens (AAAs) - IL13Rα2, Fra-1, EphA2 - and the most common molecular aberrations typical for astrocytomas as potential markers to screen the status of tumour-derived cell cultures *in vitro*.

**Methods:**

The tumour-derived cell cultures were established from high-grade astrocytomas. The expression analyses of the tested genes were performed via semi-quantitative real-time PCR and subsequently verified by immunohistochemical and immunocytochemical technique. The analyses of molecular aberrations at DNA level included gene dosage status evaluation based on real-time PCR, sequencing analysis, and loss of heterozygosity (LOH) assay.

**Results:**

The expression analyses based on semi-quantitative real-time PCR showed that in the final stage of culture the expression level of all tested AAAs was significantly higher or at least comparable to that of primary tumours; however, two expression patterns were observed during cell culture establishment. Analysis at the single cell level via immunocytochemistry also demonstrated an increase of the level of tested proteins and/or selection of tumour cell populations strongly positive for AAAs vs. other cell types including admixed non-tumoural cells. Confrontation of AAA expression data with the results of molecular analyses at DNA level seems to support the latter, revealing that the expression pattern of astrocytoma-associated antigens in tumour-derived cells in subsequent stages of culture is convergent with changes in the molecular profile of examined cell populations.

**Conclusions:**

The consistency of the obtained results seems to support the use of the selected AAAs, in particular IL13Rα2 and Fra-1, as tools facilitating the establishment of tumour-derived cultures. However, the intratumoural heterogeneity of high-grade astrocytomas may require further detailed characterisation of the molecular profile of a tumour in order to evaluate the value of the experimental model in relation to the individual context of particular studies.

## 1 Background

Tumour-derived cell cultures are a common model used for studies including tumourigenesis, signalling pathway dysregulation, mechanisms of drug resistance or the search for new therapeutic approaches.

Nevertheless, there is a controversy regarding the feasibility of development a representative model in artificial *in vitro* conditions and the utility of tumour-derived cell lines. Several recent reports discuss this issue with regard to glioma-derived cells cultured in the various experimental conditions [1]-[6].

We report here an attempt to exploit tumour-associated antigens as simple tools to characterize the status of astrocytoma-derived cell cultures and indicators of presence of neoplastic cells during cell culture establishment. Based on previously published data, astrocytoma-associated antigens (AAAs), such as Interleukin-13 Receptor α2 (IL13Rα2), Fos-Related Antigen 1 (Fra-1), and EphA2 receptor tyrosine kinase (EphA2) were selected. These antigens had been described as molecular denominators of high-grade astrocytomas and potential novel therapeutic targets [7]-[14]. Additionally, the most frequent molecular abnormalities presented in astrocytomas (LOH 10p and LOH 10q; TP 53 mutations; EGFR amplification/chromosome 7 polysomy; EGFRvIII variant; CDKN2A deletions; IDH1 mutations) were used as markers of neoplastic cells at the DNA level [15]-[18].

## 2 Results

### 2.1 Characteristics of astrocytoma tumours exploited for generation of cell cultures

Initially, seven astrocytoma samples were used to obtain the cell cultures. However, in two cases the culture growth was not effective. Astrocytoma cells derived from remaining four cases had been cultured successfully for up to 5–7 months. In a case of the newest sample, this process took up to 3–4 months. Then, all cultures were terminated. Material for further analyses was collected three times: at the early (1–2 months), middle (3–4 months) and late (5–7 months) stages of cell culture, depending on the proliferation ability of cells derived from particular tumours.

**Figure 1:**
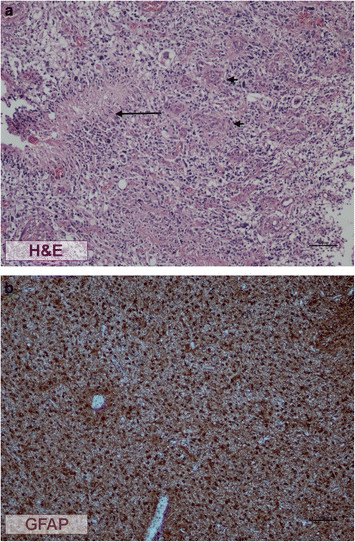
**Basic histological characteristics of representative astrocytoma sections.** Histological pattern of one of the glioblastoma cases showing cellular pleomorphism, palisade necrosis (arrow) and microvascular proliferations (short arrows) **(a)**. G112 tumour did not show any areas of necrosis or microvascular proliferations. Cells with pleomorphic nuclei and mitotic activity were strongly GFAP immunoreactive thus it was classified as WHO grade III **(b)**.

The general description of tumours including basic clinical data and histopathological characteristics were compiled in Table 1 and Figure 1.

**Table 1:** Basic clinical characteristics

**Sample**	**Histopathology**	**Age**	**Sex**	**Location**	**Radiotherapy/chemotherapy**
**G 108**	Glioblastoma	59	F	Frontal	No
**G 111**	Glioblastoma	50	F	Parieto-occipital	No
**G 112**	Anaplastic astrocytoma	58	M	Temporal	No
**G 113**	Glioblastoma	73	F	Parietal	No
**G 114**	Glioblastoma	65	M	Parietal	No

The molecular profile of tumours exploited to establish cell culture were characterised initially with regard to the most common abnormalities occurring in astrocytomas: TP 53 mutational status, EGFR copy number & chromosome 7 polysomy, EGFRvIII presence, CDKN2A status, loss of heterozygosity on 10p and 10q, and IDH1 mutational status. Table 2 presents the molecular characteristics of astrocytoma initial tumours.

**Table 2:** Molecular characteristics of initial tumour samples

**Sample**	**TP53**	**EGFR amp./ch. 7 polys.**	**EGFRvIII**	**CDKN2A**	**LOH 10p**	**LOH 10q**	**IDH1**
**G 108**	MT Ex 8, 262, GGT → GTT	A/P	-	DEL	LOH	LOH	-
**G 111**	WT	A	-	DEL	LOH	LOH	-
**G 112**	WT	A	-	DEL	LOH	LOH	-
**G 113**	WT	Low A	-	-	ROH	ROH	-
**G 114**	WT	Low A	-	DEL	NI	LOH	-

*MT* = mutation, changes in nucleotide sequences are indicated by arrows; *WT* = wild type; *A* = amplification; *P* = polysomy; low *A* = amplification at low level; *DEL* = deletion; *LOH* = loss of heterozygosity; *ROH* = retention of heterozygosity; *NI* = non informative.

**Figure 2:**
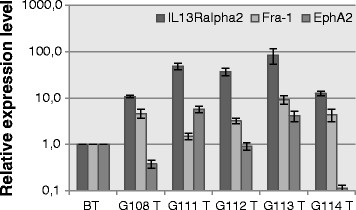
**Evaluation of Il13Rα2, Fra-1, and EphA2 expression in initial tumours at the mRNA level.** Results of real-time PCR (means ± SD) enabled the identification of tumours exhibiting overexpression of astrocytoma-associated antigens. BT = normal brain tissue; G108-G114T = particular tumour samples.

**Figure 3:**
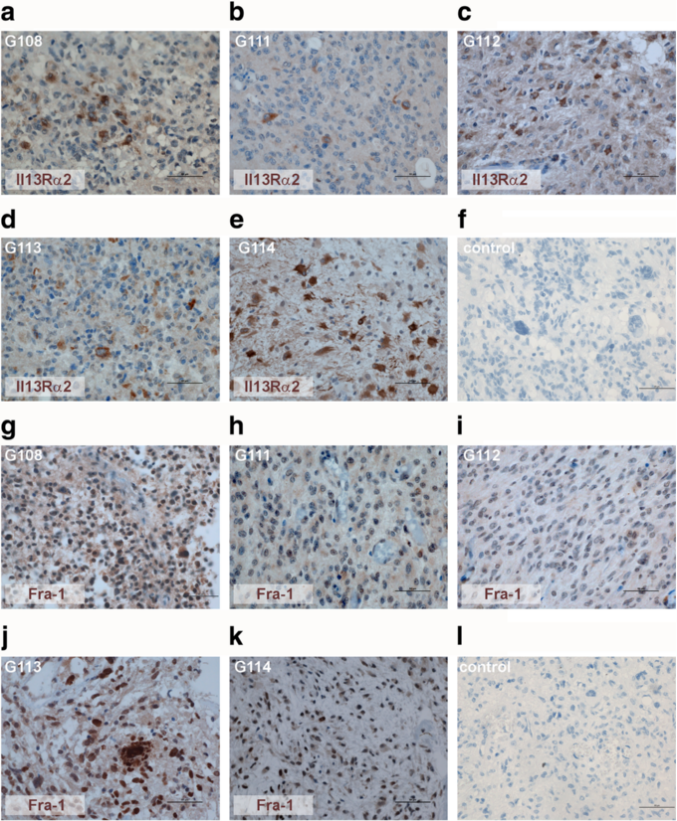
**Immunohistochemical analysis of Il13Rα2 and Fra-1 expression in initial tumours.** The IL13Rα2 immunoreactivity was observed in all tested tumour specimens **(a-e)**; Fra-1 antigen was detected in four tested samples **(g-k)**; negative controls **(f, l)**.

Moreover, the original tumours were evaluated concerning astrocytoma-associated antigens expression (IL13Rα2, Fra-1 and EphA2) using the real-time PCR method (Figure 2). In comparison to a control, which was represented by commercially available RNA from normal human brain, our results demonstrated an overexpression of IL13Rα2 in all tested samples, a significantly higher level of Fra-1 in four samples (G108, G112, G113, G114) and a significantly higher expression of EphA2 in two samples (G111, G113), (P < 0.05). One sample (G111) presented an expression of Fra-1 comparable to that reported for a normal brain. The expression of EphA2 was similar to that of a normal brain in the G112 sample and even lower in two other tested samples - G108 and G114 (P < 0.05). Additionally, immunohistochemical analyses were performed for two antigens (IL13Rα2, Fra-1) overexpressed at mRNA level in the majority of the original tumours (Figure 3).

Strong membrane and cytoplasmic reactivity for IL13Rα2 was found in three tumour sections, and weak cytoplasmic staining was observed in two other cases (Figure 3a-e). Nuclear immunoreactivity for Fra-1 was observed in three tumour sections, and one sample presented weak cytoplasmic expression pattern (Figure 3g-k).

The study at the mRNA level showed that in four of the five tested samples an overexpression of two selected astrocytoma-associated antigens occurred. The IL13Rα2 immunoreactivity was found in all tested tumour specimens, while Fra-1 antigen was detected in four tested samples.

### 2.2 Astrocytoma-derived cell cultures presented an increasing level of tumour –associated antigens

The expression of astrocytoma-associated antigens (IL13Rα2, Fra-1, and EphA2) in particular stages of astrocytoma-derived cultures was assessed both at the mRNA and protein levels.

**Figure 4:**
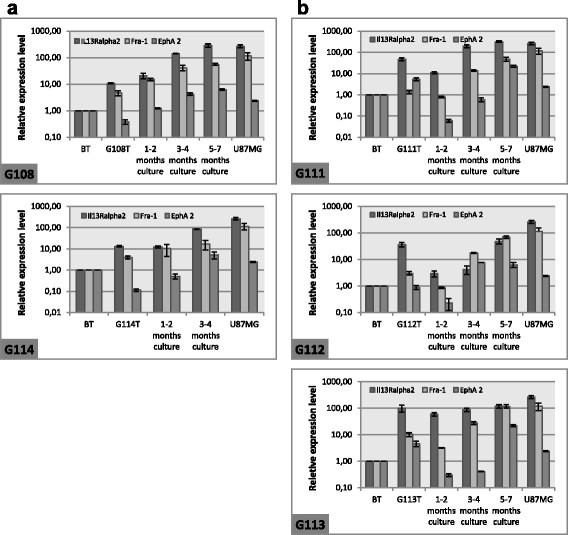
**Analysis of Il13Rα2, Fra-1, and EphA2 expression in astrocytoma-derived cell cultures at the mRNA level.** Results of real-time PCR (means ± SD) demonstrated two patterns of of AAA expression during particular stages of cell culture - a gradual increase of the AAA mRNA levels for G108 and G114 **(a)** and transient decrease of the AAA expression in the first step of culture (1-2 months) for G111, G112, and G113 samples **(b)**. U87MG was used as positive control. BT = normal brain tissue; G108T-G114T = initial tumour samples.

**Figure 5:**
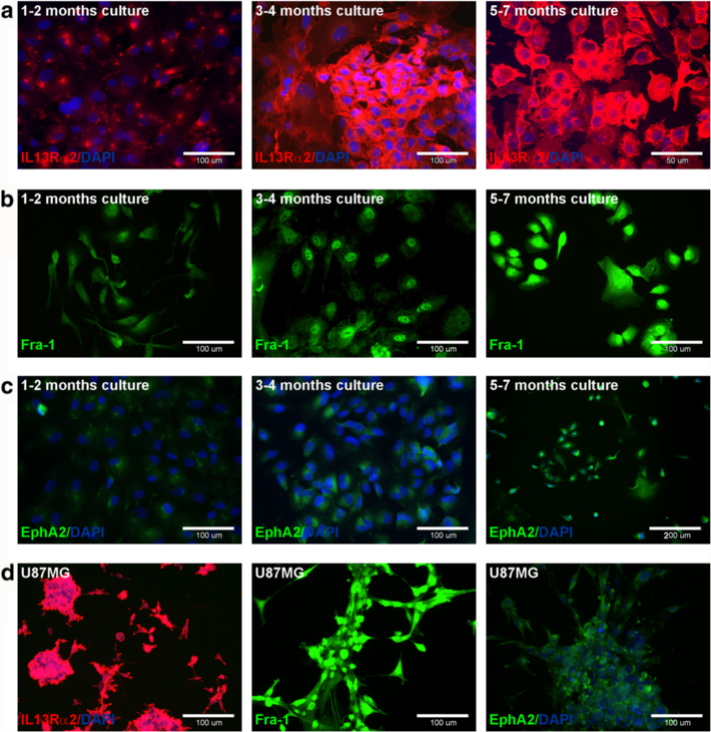
**Expression of astrocytoma-asociated antigens in the representative tumour-derived cell culture (G113).** Immunocytochemical results presenting expression of IL13Rα2 **(a)**, Fra-1 **(b)** and EphA2 **(c)** antigens in subsequent populations of astrocytoma cells derived from particular steps of culture (1–2 months; 3–4 months; 5–7 months). U87MG was used as positive control **(d)**.

The results of real-time PCR demonstrated a gradual increase in the expression of all tested astrocytoma-associated antigens during particular stages of cell culture for the G108 and G114 samples. In the final step of culture, the expression of the tested genes was significantly higher than that observed in original tumours (P < 0.05), (Figure 4a). In three of the tested cases (G111, G112, and G113), a transient decrease in the tested genes expression in the first step of culture (1–2 months) was observed in comparison to the original tumour tissue (P < 0.05). Subsequently, the expression increased, finally reaching the level observed in the original tumour or higher (P < 0.05), (Figure 4b). In one case, a fluctuation of EphA2 level was detected in the final stage of G112 culture. This observation was interpreted as a subtle downregulation of expression observed after 5–7 months of culture in comparison to the 3–4 month step (P < 0.05). However, the EphA2 mRNA level was still significantly higher than that detected in the original tumour (P < 0.05). Since real-time PCR method enables the assessment of gene expression in the whole population of tested cells, our results were verified at the single cell level by immunocytochemistry technique. The results confirmed those observed at the mRNA level, demonstrating the expansion of cell populations positive for AAAs and/or increased expression of the tested genes. Figure 5 depicts the representative results of immunocytochemistry demonstrating changes of AAA levels in the G113-derived culture. Additionally, the immunocytochemical data revealed that overexpression of the Fra-1 antigen in astrocytoma cells can be manifested by the cytoplasmic and nuclear localisation of this protein (Figure 5b). The differences observed for EphA2 protein levels seem to be more subtle than that reported for IL13Rα2 and Fra-1 (Figure 5c). This finding is consistent with real-time PCR data demonstrating the relatively low expression level of this antigen in the tested astrocytoma specimens (Figure 4).

As a positive control for AAA expression analyses both at mRNA and protein levels, the U87MG cell line was used (Figure 4, Figure 5d).

### 2.3 Utility of the selected molecular hallmarks of astrocytomas as markers of tumour cells *in vitro*

The initial molecular characteristics of astrocytoma samples enabled the selection of anomalies presented in primary tumours which could be further analysed in subsequent stages of astrocytoma-derived culture (Table 2).

**Figure 6:**
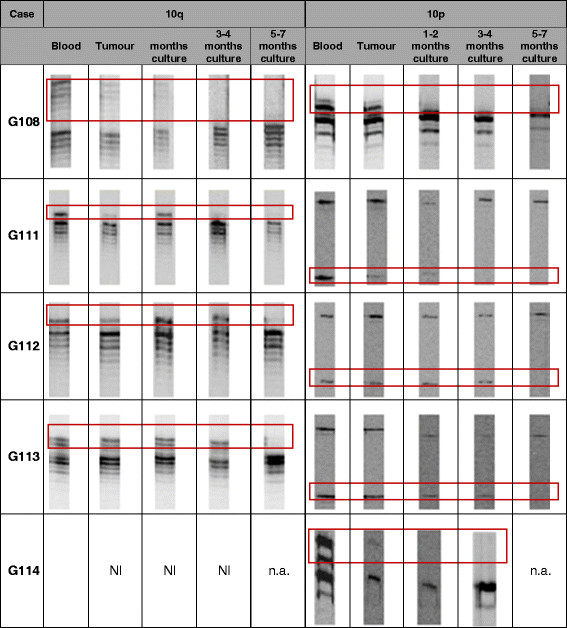
**Results of comparative analyses of LOH 10q and LOH 10p.** The comparative analysis of initial tumours vs. cells collected from particular stages of culture in relation to the loss of heterozygosity on 10p and 10q. Results of LOH assay visualized by change of the allelic signal intensity in the tumour sample compared with that in the control (rectangles); NI = non-informative; n.a. = non-analysed.

The comparative analysis of initial tumours vs. cells collected from particular stages of culture in relation to the loss of heterozygosity on chromosome 10p and 10q is presented in Figure 6. The results demonstrated that positive selection of cells harbouring these anomalies *in vitro* became visible at middle/final stages of culture. Additionally, in one case (G113), we identified LOH 10p and LOH 10q at middle/final stages of culture, while these anomalies were not detected in the original tumour.

The CDKN2A status was monitored with the use of real-time PCR at the DNA level (Table 3). The results showed that deletion of the CDKN2A gene was propagated *in vitro*. Moreover, the data revealed a gradual decrease in a gene dosage level in subsequent passages of culture (G111, G112, G114), suggesting the positive selection of cell populations with this aberration.

**Table 3:** CDKN2A status evaluated with the use of real-time PCR at the DNA level

**Sample**	**G108**	**G111**	**G112**	**G113**	**G114**
**Tumour**	DEL (0.43)	DEL (0.17)	DEL (0.52)	- (0.86)	DEL (0.35)
**1-2 months culture**	DEL (0.67)	DEL (0.35)	- (1.22)	- (1.32)	DEL (0.05)
**3-4 months culture**	DEL (0.76)	DEL (0.00)	DEL (0.64)	- (0.89)	DEL (0.00)
**5-7 months culture**	DEL (0.54)	DEL (0.02)	DEL (0.00)	- (0.95)	n.a.

Results are presented as relative gene dosage levels in comparison to control (values in brackets). Values ≤ 0.85 were considered as detection of a cell population demonstrating CDKN2A deletion (details in Methods section). *DEL* = deletion; *n.a*. = non-analysed.

The analysis of EGFR gene dosage status via real-time PCR revealed a decline in EGFR amplification *in vitro* to a very low level that was closed to the threshold value assumed for this method (Table 4). EGFR gene overdosage resulting from chromosome 7 polysomy was detected in the G108 sample both in initial tumour and subsequent passages of culture. In one case (G114), a relatively stable, but low level of EGFR gene overdosage *in vitro* was observed, despite the fact that chromosome 7 polysomy was not identified in this sample.

**Table 4:** EGFR gene dosage status evaluated with the use of real-time PCR

**Sample**	**G108**	**G111**	**G112**	**G113**	**G114**
**Tumour**	A/P (18.50)	A (6.18)	A (9.25)	Low A(1.78)	Low A (2.02)
**1-2 months culture**	Low A/P (3.58)	- (1.12)	Low A(1.69)	- (1.00)	Low A (3.57)
**3-4 months culture**	Low A/P (2.24)	Low A (1.58)	- (1.02)	- (1.31)	Low A (2.97)
**5-7 months culture**	Low A/P (1.85)	Low A (1.62)	Low A (1.68)	Low A(1.83)	n.a.

Results are presented as relative EGFR gene dosage levels in comparison to control (values in brackets). Values ≥ 5 were considered as EGFR amplification (A); values 1.5-5 were considered as low amplification level of EGFR (low A). Polysomy 7 (P) were examined based on GPER gene dosage status (details in Methods section). n.a. = non-analysed.

Mutational analysis of the TP53 gene revealed a mutation in the G108 tumour propagated along with all steps of culture. In two cases (G113, G114), TP53 mutations were detected *in vitro* only in all stages of cell culture (Table 5).

**Table 5:** TP 53 mutational status

**Sample**	**G108**	**G111**	**G112**	**G113**	**G114**
**Tumour**	MT ex 8, c 262, GGT → GTT	WT	WT	WT	WT
**1-2 months culture**	MT ex 8, c 262, GGT → GTT	WT	WT	MT ex 7; c 245 GGC → AGC	MT ex 7; c 248 CGG → CAG
**3-4 months culture**	MT ex 8, c 262, GGT → GTT	WT	WT	MT ex 7; c 245 GGC → AGC	MT ex 7; c 248 CGG → CAG
**5-7 months culture**	MT ex 8, c 262, GGT → GTT	WT	WT	MT ex 7; c 245 GGC → AGC	n.a.

*MT* = mutation, changes in nucleotide sequences are indicated by arrows; *WT* = wild type; *ex* = exon; *c* = codon; *n.a*. = non-analysed.

## 3 Discussion

Interleukin-13 Receptor α2, Fos-Related Antigen 1, and EphA2 receptor tyrosine kinase are considered as astrocytoma-associated antigens (AAAs) and potential therapeutic targets [7]-[14]. In this paper, the utility of these proteins as potential markers facilitating the monitoring of the status of astrocytoma-derived cell cultures *in vitro* was determined. Initial evaluation, performed by a semi-quantitative real-time PCR method, revealed that all examined tumours displayed overexpression of at least one of the selected astrocytoma-associated antigens and four of the five tested samples demonstrated a significantly higher level of two of AAAs in comparison to that reported for a normal brain. The analyses verified subsequently at the protein level showed the IL13Rα2 immunoreactivity in all tumour specimens, and Fra-1 antigen in four of the five examined samples. The results obtained in this study are consistent with previously reported immunohistochemical findings [7]. Moreover, comparative analyses of AAA levels during particular stages of cell culture were also performed in the present study. The results of analyses at the mRNA level showed that the expression of all tested AAAs in the final stage of culture was significantly higher or comparable to the levels detected in primary tumours. However, two expression patterns during cell culture establishment were observed. Additionally, the expression of AAAs at the single cell level was examined. Immunocytochemistry results demonstrated an increase of the tested proteins level and/or a selection of cell populations strongly positive for AAAs vs. astrocytoma cells presenting a lower expression of them and/or admixed non-tumoural cells. Discrepancies in AAA levels between glioblastoma xenograft tumours and xenograft-derived cell lines were also noticed by Wykosky at al., who explained this phenomenon as a consequence of interaction with other cellular signalling pathways [7].

The simplest explanation of such a difference is the proposal that artificial conditions affect the expression levels of examined genes *in vitro*; however, such an approach does not include the phenomenon of cell selection [19],[20]. To verify this hypothesis, analyses based on DNA markers were performed. Molecular anomalies typical for astrocytomas (TP 53 mutations, EGFR gene overdosage, CDKN2A deletion; LOH 10p, LOH 10q) identified in original tumours used for the generation of cell cultures were applied as potential markers of tumour cells. In accordance with previous reports, EGFR overdosage resulting from gene amplification was either almost completely eliminated *in vitro* or retained in culture at very low levels. The reasons for this phenomenon are still under investigation, as it is the possibility of the retention of this aberration in culture [21]-[24]. These facts made doubtful the use of this aberration as a potential marker of tumoural cells *in vitro*.

Comparative analyses of the loss of heterozygosity on 10p and 10q, CDKN2A deletion and TP53 mutations suggest the positive selection of the cells harbouring these anomalies in culture vs. other cells including possible admixed non-tumoural cells. However, in G111 and G112 cases, we observed that some anomalies (LOH 10p, LOH 10q) previously identified in initial tumours were not detected in the first stages of cell culture establishment. We hypothesised that it could be caused by intratumoural heterogeneity and/or an admixture of non-neoplastic cells which could be selected positively vs. tumour cells at the beginning of tumour culture generation *in vitro*[25]-[27].

Parallel analysis of the examined molecular markers and AAA expression data revealed that the reduction of AAA level observed at the beginning of culture was coincident with a depletion of the cell population harbouring the tested molecular anomalies. Consequently, the increasing expression of AAAs was accompanied by propagation of molecular aberrations in culture suggesting the positive selection of tumoural cells vs. other cell types.

One of the potential options is the presence of GASCs (GB-associated stromal cells) - a novel population of cells detected in the glioblastoma microenvironment [28]. Our initial results with the use of putative markers of GASCs did not exclude the possibility that this population of cells could be expanded periodically vs. astrocytoma cells *in vitro* (data not shown). Notwithstanding this hindrance, in the final stage of culture the positive selection of tumour cells harbouring aberrations typical for astrocytomas in all the tested samples was observed.

In the majority of cases, the examined molecular anomalies were reported in original tumours and further propagated in culture. However, some anomalies were detected exclusively in culture in the following samples: G113, G114 – TP53 mutations, G113 – LOH 10p, LOH 10q. Two possibilities are considered here. First, this is an artifact generated *in vitro.* Second, these anomalies were initially present only in a small percentage of cells in tumour *in situ* below the level of detection. The latter seems to be supported by the fact that these anomalies normally occur in astrocytomas [15],[18]. A similar dilemma was faced by Solomon et al., who found no evidence of artifactual genetic alterations in tumour cells caused by *ex vivo* culture. Moreover, they stressed that some of these genetic abnormalities had been previously identified in tumour-derived cell lines and subsequently their presence were confirmed *in vivo*[27]. Nickel et al. also demonstrated that intratumoural heterogeneity may result in false-negative data for aberrations that appear only in a minority of cells in a sample [29].

Such reports may contribute to a re-evaluation of the assumption that tumour-derived cell cultures are a non-valuable experimental tool, due to the risk of the accumulation of spurious genetic alterations. Moreover, some studies presented this assertion based on a comparison of commercially available cell lines vs tumour samples obtained from other patients [30]-[33].

In contrast, Yost et al. reported the utility of previously validated glioblastoma-derived cultures as pre-clinical models in defining tumour genetic predictors of drug response and the escape of tumours from selective pressures (e.g. treatments) [21]. This point of view seems to be consistent with the findings suggesting that clonal selection is crucial in the recurrence of gliomas that, due to their intratumoural heterogeneity, often gain resistance to treatment and rapidly recur. Furthermore, the molecular profiles of recurrent gliomas can be different from the characteristics of primary tumours resulting in more aggressive phenotype [34]-[38]. This scenario seems to resemble the situation observed *in vitro* when some differences between molecular profile of initial tumour and tumour-derived culture were identified.

## 4 Conclusions

Our results revealed that the expression pattern of astrocytoma-associated antigens in original tumours and subsequent stages of cell culture is consistent with the molecular profiles of the examined cell populations. This finding justifies the utilization of IL13Rα2, Fra-1, and EphA2 as tools to monitor of the status of astrocytoma-derived cultures and putative markers of neoplastic cells presence *in vitro*. However, the relatively low/moderate expression of EphA2 in the majority of tested samples seems to minimize the importance of this antigen as a potential marker of tumoural cells *in vitro*.

The results of our long-term experiments demonstrated a dynamic of changes in cell culture *in vitro* and revealed periodical absence/reduction of the selected markers present initially in a tumour *in situ* (in three of the five tested samples). This phenomenon could be caused by overgrowth of non-tumoural cells in the first stages of culture *in vitro*. Subsequently, stabilization of selected markers at high level was observed indicating the domination of tumoural cells in the later stages of culture. Our results suggest that longer culture can reduce the risk of non-neoplastic cells overgrowth, but at the expense of initial heterogeneity maintenance. Due to the intratumoural heterogeneity of high-grade astrocytomas, further molecular characterisation may be required in order to evaluate the usefulness of astrocytoma-derived culture as an experimental model *in vitro*, dependent on the purpose of the individual study (e.g. the level of therapeutic targets).

## 5 Methods

### 5.1 Astrocytoma cell culture

Tumour samples were obtained from patients who underwent neurosurgery at the Department of Neurosurgery, Medical University of Lodz, Poland. All samples were collected under protocols approved by the ethical committee of the Medical University of Lodz.

Tumour tissue was minced in cell culture media and passed through a cell strainer (40 μm; BD Falcon™) to obtain a single cell suspension. Cells were washed with PBS and seeded in T25 cell culture flasks or 6-well plates. Subsequently, the cells were cultured in DMEM/F12 media supplemented with 10% FBS, 2 mM L-glutamine (PAA), and antibiotics (Sigma-Aldrich). Depending on proliferation activity, the cells were passaged to a new culture dish every 3–10 days and expanded for subsequent analyses. Further analyses were performed with the use of cells obtained during three periods of culture - early (1–2 months), middle (3–4 months) and late (5–7 months). Additionally, U87MG cells were used as a control astrocytoma (WHO IV) cell line.

### 5.2 Analyses of molecular markers at DNA level

#### 5.2.1 Quantitative real-time PCR at DNA level - EGFR gene dosage status

To determine the EGFR gene dosage level in original tumour tissue and astrocytoma cells in culture, quantitative real-time PCR was performed using a Rotor-Gene 6000 instrument (Corbett Life Science). The following EGFR primers were used for amplification: F: AACCATGCCCGCATTAGCTC; R: AAAGGAATGCAACTTCCCAA.

Each sample was amplified in triplicate in a reaction volume of 10 μl containing 20 ng of DNA, KAPA SYBR FAST Universal 2X qPCR Master Mix (Kapa Biosystems) and forward and reverse primers. The cycling conditions were performed according to the manufacturer’s protocol. RNase P was used as a reference gene for normalization of the target gene dosage level. To confirm the specificity of the amplification signal, the gene dissociation curve was considered in each case. Utilizing the method described previously by Pfaffl et al. [39], the normalized relative EGFR gene dosage level of the tested samples was calculated versus the control sample, based on each sample’s average CT value and each gene’s average PCR efficiency. As a control sample, DNA derived from non-tumoural tissue (leukocytes) was used, on the assumption that the gene dosage in normal tissue would be 1. To distinguish between the amplification of EGFR vs. chromosome 7 polysomy, the method described and validated previously was applied [40]. Briefly, the technique presented is based on the assumption that the ratio of EGFR to the other marker located within chromosome 7 (GPER; 7p22) would be equal to 1, if there are no amplicons (assuming no LOH within either of the analysed loci). The following method of result interpretation was applied: the cumulative EGFR gene dosage was assessed using the ratio of EGFR to RNaseP (RPP25; 15q24.2); chromosome 7 polysomy was identified when the ratio of GPER to RNaseP was higher than 1.5; while EGFR amplification was identified when the ratio of EGFR to GPER was higher than 1.5.

#### 5.2.2 Quantitative real-time PCR at DNA level – detection of CDKN2A deletion

To determine the CDKN2A deletions, quantitative real-time PCR reactions were performed as described above. The primer data were based on previous report [40]. The reference gene was RNaseP. Each sample was analysed three times. DNA derived from non-neoplastic tissue (leukocytes) was used as a control. To determine the threshold value for the tested gene, similar analyses were performed for 10 samples of DNA obtained from leukocytes. Values equal to/less than 3σ the mean were considered to represent gene dosage reduction and the presence of cells presenting gene deletion. Additionally, the results were verified via agarose gel electrophoresis using BioRad Quantity One 1-D Analysis Software.

#### 5.2.3 TP53 and IDH1 sequencing analysis

Exons 5–8 of the TP53 gene and exon 4 of the IDH1 gene were amplified by PCR as previously described and sequenced using the dideoxy termination method and the SequiTherm Excel DNA Sequencing kit (Epicentre Technologies) and LiCor automated sequencer according to the manufacturer’s protocol. Sequencing analysis was performed at DNA and cDNA level as described before [40],[41].

#### 5.2.4 LOH assay

LOH analyses were performed using paired tumour specimens and corresponding peripheral blood samples. The following LOH markers were used: D10S1709, D10S587 (10q); D10S189, D10S1172 (10p). Forward primers were 5′-end fluorescence-labelled. PCR was performed in thermocycling conditions individually established for each pair of primers. PCR products were denatured and gel electrophoresis in an LiCor automatic sequencer system was applied to the separation and analysis of PCR-generated alleles. The analysis of the LOH results was based on the reduction of the allelic signal intensity in the tumour sample compared with that in the control.

### 5.3 Expression analysis

#### 5.3.1 Quantitative real-time RT-PCR

Reverse transcription was performed using a QuantiTect reverse transcription kit (Qiagen) according to the manufacturer’s protocol. Real-time PCR was performed using a RotorGene 6000 instrument (Corbett Life Science). IL13Rα2, Fra-1, and EphA2-specific primers were used for amplification of the tested genes (IL-13R2, F ACTGGTATGAGGGCTTGGAT, R TCTGATGCCTCCAAATAGGG; Fra-1, F GCCCACTGTTTCTCTTGAGC, R GGAGATAGGGTTGGGTGGAT; EphA2, F GTGTACAAGGGCATGCTGAA, R AACTTGTCCAGGGCCCCATT). GUSB was used as a reference gene for normalization of the target gene expression level. Each sample was amplified in triplicate in a reaction volume of 10 μl containing 20 ng of cDNA, KAPA SYBR FAST Universal 2X qPCR Master Mix (Kapa Biosystems) and forward and reverse primers. The cycling conditions were performed according to the manufacturer’s protocol. To confirm the specificity of the amplification signal, the gene dissociation curve was considered in each case. Normalized relative expression levels for the examined genes in the tested samples versus the control sample were calculated utilizing the method described previously by Pfaffl et al., based on each sample’s average CT value and each gene’s average PCR efficiency [39]. RNA derived from a normal human brain (Total RNA, Brain, Human; Agilent Technologies) was used as a control.

#### 5.3.2 RT-PCR

Reverse transcription was performed using QuantiTect reverse transcription kit (Qiagen) according to the manufacturer’s protocol. The conventional RT-PCR was applied to examine the tested tumour samples in terms of EGFRvIII expression. The results were obtained with the use of two pairs of primers described previously [42], and visualized on agarose gel; RNA derived from tumour tissue positive for EGFRvIII was used as a control.

#### 5.3.3 Immunocytochemistry

For immunocytochemical analysis, cell cultures were fixed for 15 min in 4% paraformaldehyde in PBS (and permeabilized with 0.1% Triton X-100 for 10 min, if necessary). Non-specific binding sites were blocked with 2% donkey serum in PBS for 1 h. Subsequently, the cells were incubated for 1 h with the following antibodies: anti-IL13 receptor alpha 2 (ab55275, Abcam); anti-Fra-1 (sc-605, Santa Cruz Biotech.); anti-EphA2 (sc-924, Santa Cruz Biotech.). For visualization, the appropriate species-specific fluorochrome-conjugated secondary antibodies (1:500, donkey anti-rabbit AlexaFluor488, 1:500, donkey anti-mouse Alexa-Fluor594; Molecular Probes) were applied for 1 h in the dark. Controls, with secondary antibodies alone and with matched isotype controls in place of primary antibodies, were processed in the same manner. Slides were mounted with ProLongGold Antifade Reagent with DAPI (Molecular Probes), coverslipped and examined using an Olympus BX-41 fluorescence microscope.

#### 5.3.4 Immunohistochemistry

For immunohistochemistry 5-um thick sections of formalin-fixed and paraffin-embedded tissue were used with the following antibodies: anti-IL13 receptor alpha 2 (1 μg/ml; ab55275, Abcam), anti-Fra-1 (1:200; sc-28310, Santa Cruz Biotech.). Epitope retrieval method included citrate buffer pH 9 and microwave heating for Fra-1 analysis and citrate buffer pH 9 with water bath heating for IL13Rα2 detection.

For visualization Dako EnVision + System-HRP (DAB) for Use with Mouse Primary Antibodies, (K4007) kit was used. Negative controls were paralleled sections treated as above with omission of the primary antibody.

### 5.4 Statistical analysis

The real-time PCR data were expressed as means ± SD. The data for initial tumour samples were analyzed with the use of the Student t test. For further analyses, when more than two groups were compared, the Kruskal–Wallis test was used initially to identify a difference and, if this proved significant, individual groups were further investigated using the Conover–Inman posthoc test. In all tests, P < 0.05 was considered significant.

## Competing interests

The authors declare that they have no competing interests.

## Authors’ contributions

MWP designed the project, performed the cell cultures, carried out the expression analyses at mRNA and protein level and performed final analysis of obtained data. MZ performed the cell cultures and participate in critical revision of manuscript. MS carried out the analyses at DNA level. WP performed histopathological classification of samples. DJJ was responsible for samples protection and collection. BS was responsible for immunohistochemical analyses and critical revision of manuscript. PPL participated in the samples selection, coordination of the project and critical revision of manuscript. All authors have been involved in drafting the manuscript. All authors read and approved the final manuscript.
